# Modeling the effect of loving pedagogy dispositions and teacher self-efficacy on teacher burnout

**DOI:** 10.3389/fpsyg.2023.1157324

**Published:** 2023-05-12

**Authors:** Shuya Chen

**Affiliations:** Basic Courses Teaching Department, Henan Judicial Police Vocational College, Zhengzhou, Henan, China

**Keywords:** loving pedagogy, teacher burnout, teacher self-efficacy, EFL, SEM

## Abstract

**Introduction:**

Foreign language teaching is a demanding and challenging profession, and teacher burnout is a common issue in this field. There is a growing research interest in exploring the factors that can protect teachers from burnout and promote their well-being, as well as their effectiveness in the classroom. One such factor might be loving pedagogy, which refers to a teacher’s positive and compassionate attitudes and behaviors toward their students. This study aimed to examine the association between Dispositions toward Loving Pedagogy (DTLP), teacher self-efficacy, and teacher burnout among a sample of Chinese English as a foreign language (EFL) teachers.

**Methods:**

The participants included 428 English teachers from various parts of China. Data on the three constructs were gathered using an electronic survey which comprised three valid questionnaires for these variables. Structural equation modeling (SEM) was used to test the hypothesized relations among the latent constructs.

**Results:**

The results indicated that loving pedagogy dispositions negatively affected teacher burnout and that teacher self-efficacy mediated the effect of loving pedagogy on burnout. More precisely, higher levels of loving pedagogy were associated with greater levels of teacher self-efficacy, which is in turn negatively affected teacher burnout.

**Discussion:**

These outcomes shed more light on the importance of loving pedagogy dispositions for teachers’ mental health and well-being. The findings have implications for theory and practice, as they suggest that fostering loving pedagogy dispositions among teachers can help prevent burnout and promote their well-being. Teacher training programs could integrate this construct into their curricula to support teachers in developing these attitudes and behaviors. Additionally, future research could explore ways to enhance loving pedagogy and self-efficacy among teachers and assess their impact on teacher well-being and effectiveness.

## 1. Introduction

Burnout is conceptualized as “a psychological syndrome emerging as a prolonged response to chronic interpersonal stressors on the job” (Maslach and Leiter, 2016, p. 103). The concept of burnout is usually shaped when one finds work unsatisfactory, frustrating, and unrewarding and is reflected by three facets, namely exhaustion, lack of personal accomplishment and effectiveness, and feelings of cynicism toward and detachment from work (Janssen et al., 1999; Maslach et al., 2001). Recognized as the core aspect of burnout (Taris et al., 2005), emotional exhaustion is a physical depletion or the feeling of being emotionally worn-out that results from inordinate job demands (i.e., work overload, and cognitive demands) and constant exposure to stress (Wright and Cropanzano, 1998; Wullur and Werang, 2020). It is worth pointing out that a person who is regularly exposed to stressors, fatigue, aggressiveness, discouragement, inefficacy, discomfort, and restriction is more inclined to experience burnout (Park and Shin, 2020). Moving to burnout among teachers, research in the past two decades has always indicated that teaching is one of the most inherently stressful occupations and that stress brought about by work-related demands can be a predictive factor of teachers’ emotional exhaustion and burnout (Alarcon, 2011; Xu, 2019; Schaack et al., 2020; Skaalvik and Skaalvik, 2020). Teacher burnout is often a result of job strain and considering the demands of the occupation, those teachers with less coping competencies are more likely to fall prey to burnout (Taris et al., 2001; Santavirta et al., 2007; Zhang et al., 2019). As Madigan and Kim (2021a) asserted, given that burnout is often the cause behind teacher attrition, it is of critical significance in teacher preparation programs. Indeed, previous research has shown that burnout can be a prime contributor to teacher attrition and turnover (Rumschlag, 2017; Lee, 2019; Boamah et al., 2022; Räsänen et al., 2022). As demonstrated by previous research, teacher burnout can have adverse effects not only on teachers’ well-being and mental health, such as lower engagement (Hakanen et al., 2006; Salmela-Aro et al., 2019), higher levels of depression (Steinhardt et al., 2011; Capone et al., 2019), low levels of job satisfaction (Smetackova et al., 2019; Capone and Petrillo, 2020), but also on learners’ achievement (Madigan and Kim, 2021b), and motivation (Zhang and Sapp, 2008; Shen et al., 2015).

Given the fact that teacher burnout has negative personal and interpersonal implications for both teachers and students, research on this concept requires more attention. In addition, since teaching is a critical occupation which can significantly contribute to society, it is essential to find ways to retain teachers and stop them from leaving the profession. Recognized as one of the significant causes of teachers’ turnover, burnout has been, to a greater to a lesser degree, subject to a number of investigations in various educational settings, namely second/foreign language (L2) context (e.g., Javadi, 2014; Shirazizadeh and Karimpour, 2019; Roloff et al., 2022). Nevertheless, empirical evidence on this concept is still fairly limited in educational settings, particularly in EFL contexts. Therefore, care should be exercised to explore teacher burnout and its potential precursors to alleviate this concept in EFL classrooms. Consequently, researchers have tried to addressed burnout among teachers and antecedents leading to this negative concept (e.g., Castillo-Gualda et al., 2019; Ismail et al., 2020; Maor and Hemi, 2021). Their findings indicated that there are a number of reasons involved in causing teachers to experience burnout while teaching, namely self-efficacy beliefs (Fathi et al., 2021).

In the last decade there has been a growing interest among researchers, particularly L2 researchers to investigate the self-efficacy of teachers. The empirical research being undertaken so far highlights the fact that teacher self-efficacy has a significant role in predicting both teacher performance and student learning. Teacher self-efficacy has been found to have a positive influence on learner achievement (Wang L., 2022), learners’ self-efficacy (Corkett et al., 2011), teachers’ job stress (Skaalvik and Skaalvik, 2017; Samfira and Paloş, 2021), and teachers’ commitment to the profession (Chesnut and Burley, 2015). Additionally, empirical evidence shows that teacher self-efficacy is negatively associated with teacher burnout (e.g., Skaalvik and Skaalvik, 2010; Bing et al., 2022). As it is obvious, teachers’ level of self-efficacy can be a significant determinant of teacher burnout. Hence, in the current study, I south to explore teacher self-efficacy as a potential predictor of teacher burnout.

According to Maslach et al. (2001), previous research on burnout has mainly focused on organizational factors as the predictors of this concept. Nevertheless, researchers have failed to give proper attention to affective and personality factors like loving pedagogy in burnout studies, particularly in the EFL context. Dispositions toward loving pedagogy (DTLP) pertains to teachers’ concern, sensibility, and empathy toward their learners’ growth, needs, and experiences (Zhao and Li, 2021). It has been indicated by literature that DTLP can be conducive to learners’ motivation, autonomy, and achievement, as well as to teacher engagement and well-being (Derakhshan et al., 2022; Wang et al., 2022). Consequently, given its significance in educational system, specifically L2 learning and teaching, DTLP is another variable examined in the current study.

Taken together, despite the growing number of studies examining teacher burnout, there is still a need for research exploring this construct in the EFL context. Regarding the construct of loving pedagogy, scant attention has been paid to this teacher characteristic in educational settings (i.e., EFL context). Given the limited understanding of the relationship between loving pedagogy, teacher self-efficacy, and teacher burnout in EFL contexts, there is a need to examine these relationships in greater detail. The investigation of this specific group of teachers (i.e., EFL instructors) is crucial as teaching EFL is unique and requires specific competencies and pedagogical approaches (Broughton et al., 2002). Furthermore, the study context (China) is significant because it has one of the largest populations of English learners globally, and Chinese EFL teachers often face unique challenges and experiences (Fan et al., 2021). Also, to the best of our knowledge, so far, no study has explored the effect of loving pedagogy on teachers’ burnout. As an attempt to fill the identified lacuna, the current research examines the predictability of loving pedagogy dispositions and teacher self-efficacy on EFL teachers’ burnout. Indeed, the current study examines for the very first time how EFL teachers’ self-efficacy, DTLP, and burnout are simultaneously correlated. This research is of significance as it sheds light on the importance of DTLP for promoting teacher well-being and effectiveness, and provides a deeper understanding of the mechanisms through which DTLP can impact teacher burnout.

## 2. Literature review

### 2.1. Teacher burnout

Emerged as a psychological construct in 1970s, Freudenberger (1974) first introduced burnout in 1974 to talk about the psychological condition of employees at drug addicted clinic to show how they were no longer committed to their job after working a year. Moreover, Freudenberger (1980) examined the employees’ psychological and physical status and demonstrated that they were experiencing a “state of fatigue or frustration brought about by devotion to a cause, way of life, or relationship that failed to produce the expected reward,” p. 13. As mentioned earlier, burnout is often characterized with three dimensions, namely emotional exhaustion, cynicism or depersonalization, and low efficacy or personal accomplishment (Maslach et al., 2001). *Emotional exhaustion* has to do with to the stress dimension of burnout, and is accompanied by reduced level of energy, and fatigue. Accompanied by one’s decreased emotional resources, *Depersonalization* pertains to a person’s development of the cynical and negative attitudes, and being detached from various aspects of his job. This dimension o burnout refers to the interpersonal relationship, and points to one’s emotional and cognitive alienation from himself and his colleagues. *Low efficacy/personal accomplishment* is conceived as an individual’s sense of depression, demoralization, low self-efficacy and interpersonal relationships, and a lack of achievement and productivity while working (Maslach, 2003; Taris et al., 2005; Kim et al., 2009; Maslach, 2015; Akin, 2019). According to Llorens-Gumbau and Salanova-Soria (2014), burnout is likely to develop when employees experience chronic job demands or stressors, which eventually can lead to their reduced energy resources and result in to burnout. Burnout has found its ways into various occupations and fields, such as teaching profession. As Kyriacou (2015) asserted, teacher burnout refers to “a syndrome of physical, emotional, and attitudinal exhaustion toward one’s work, which results from experiencing teacher stress over a long period” (p. 72). Teachers who experience burnout are inclined to perceive themselves negatively, hold the belief that they are not able to effectively do an important job, and have negative feelings about their learners and or peers (Chang, 2009; Gómez-Domínguez et al., 2022; Zhang et al., 2022).

Research evidence reveals that teacher burnout can predict both learner and teacher health, as well as teachers’ well-being and job satisfaction. For example, Sabancı (2009) showed that teacher burnout and its dimensions (i.e., emotional exhaustion, personal accomplishment and depersonalization) correlated negatively with organizational health. In another study, Hakanen et al. (2006) teacher burnout was negatively associated with teachers’ health and work ability. Collecting data from a sample of 313 Finish teachers, Räsänen et al. (2022) demonstrated that teacher burnout was negatively related with teacher commitment and positively with their turnover intention. In an EFL context, Wang Z. (2022) investigated the relationship between teacher burnout and learner outcomes. Their findings demonstrated that burnout of L2 teachers had a negative effect on students’ academic achievement.

Empirical evidence indicates that teachers experience burnout not only due to setbacks within the educational context, like demanding class management (Friedman, 2013), discipline problems (Chang, 2013), work-related stress (Burke et al., 1996), but also because of teachers’ own psychological factors (Talmor et al., 2005; Tsang et al., 2022). Moreover, it has been revealed that teachers’ positive psychological tendencies can reduce the negative effects of burnout and prevent it from happening in the realm of education in general and EFL context in particular (Richards et al., 2016). One such factor that can act as a defense mechanism against EFL teachers’ burnout is self-efficacy.

### 2.2. Teacher self-efficacy

It is argued that compared to objective realities, attitudes and beliefs of individuals have more significant impact on their life (Bandura, 1977; Schunk, 1984). As Bandura (1986) asserted, self-efficacy has to do with a person’s belief in his abilities and pertains to “people’s judgments of their capabilities to organize and execute courses of action required to attain designated types of performances,” p. 391. Self-efficacy refers to one’s belief toward what he/she is capable of doing rather than the judgments about his/her attributes, which are the aspects of self-concept (Schunk, 1994; Schwarzer and Fuchs, 1996). Conceptualized as a multidimensional and context-specific construct (Bong and Skaalvik, 2003), self-efficacy beliefs are skill/domain specific and there is no all-purpose measure of this concept (Pajares, 2003; Chao et al., 2019). According to Bandura (1977, 1986), self-efficacy is grounded in the social-cognitive theory and can be influenced by an individuals’ behavioral, social, affective, and psychological characteristics. Based on this theory, self-efficacy shapes individuals’ objectives and behaviors and is affected by the surrounding environment (Mughal et al., 2022). Moreover, self-efficacy is viewed to be rooted in the concept of human agency (Bandura, 2001) and can exert a strong influence on an individual’s continuous effort toward accomplishing his objectives (Zimmerman, 1995). Self-efficacy is commonly viewed as a person’s positive self-evaluation regarding his capacity to effectively control his surrounding environment and successfully have an impact upon it (Welch and West, 1995; Schyns and Von Collani, 2002). As Pintrich et al. (1993) demonstrated, carrying out a task is significantly influenced by one’s level of self-efficacy rather than his actual skills. According to Bandura (1977), there are four prime causes of self-efficacy, namely enactive mastery experiences, vicarious experiences, verbal persuasion, and physiological responses. Among which, enactive mastery experiences are often regarded as the central cause of individuals’ self-efficacy, particularly of teachers (Gale et al., 2021). Moving to the self-efficacy of teachers, teacher self-efficacy relates to teachers’ believing in their capabilities in order to bring about desired results, such as promoting students’ engagement and academic achievement (Friedman and Kass, 2002; Marschall and Watson, 2022; Yoon and Kim, 2022). Teacher self-efficacy is recognized as an instructor’s confidence in his own abilities to successfully execute teaching practices. Conceived as context-specific construct, teacher self-efficacy is concerned with teachers’ beliefs regarding their capacities to provide learners with varying amounts of help and support and have a significant effect on their and engagement (Dellinger et al., 2008; Klassen and Chiu, 2010; Tschannen-Moran and Johnson, 2011; Xiyun et al., 2022). Teachers’ beliefs toward their level of expertise, skills and mastery can help them to effectively deal with teaching-related setbacks and challenges (Sela-Shayovitz and Finkelstein, 2020). As Cherniss (1993) maintained, teacher self-efficacy contains accomplishing professional goals, improving professional development, developing well-organized teaching practices, applying approaches and procedures, having the commitment to school and teaching, being able to identify school-specific needs, and enhancing the well-being of learners (Althauser, 2015; Lauermann and König, 2016; Ford et al., 2017; Schwab et al., 2022). Jerrim et al. (2023), however, challenged the widely held belief that teachers with high self-efficacy are more effective at enhancing learner achievement by reporting that there was no evidence of a relationship between the two variables. As Tschannen-Moran and Hoy (2001) indicated, teacher self-efficacy consists of three sub-categories: teachers’ efficacy in classroom management which has to do with teachers believing in their own capacities in order to manage the classroom and control the behaviors of students, teachers’ efficacy in student engagement which is conceived as teachers’ beliefs regarding their capabilities to engage their students in the class activities, and teachers’ efficacy in instructional strategies which is about teachers’ perceptions in their abilities to plan successful teaching strategies in order to address the learning requirements.

Literature on teacher self-efficacy has constantly demonstrated that it has positive effect on both teachers’ well-being and learners’ positive outcomes (Skaalvik and Skaalvik, 2010; Burić and Macuka, 2018; Kasalak and Dagyar, 2020; Kim and Burić, 2020; Wray et al., 2022), specifically in the context of EFL (e.g., Ghonsooly and Ghanizadeh, 2013; Zonoubi et al., 2017; Fathi et al., 2021; Gao et al., 2022). Gathering data among a sample of 2,249 Norwegian teachers, Skaalvik and Skaalvik (2010) tested a relationship between burnout and self-efficacy of teachers. Analyzing the data by means of structural equation modeling, their findings demonstrated that teachers’ level of self-efficacy was negatively related to their burnout. By the same token, in the context of Norway, Skaalvik and Skaalvik (2007) examined the correlation between teacher self-efficacy and burnout. The results revealed that teachers’ self-efficacy was significantly and negatively associated with teachers’ burnout. Hassan and Ibourk (2021) investigated the association between self-efficacy, burnout and job satisfaction of teachers. Collecting data among 404 teachers, the authors revealed that there was a negative correlation between burnout and self-efficacy. Moreover, it was found that teacher self-efficacy had a positive relationship with teachers’ job satisfaction. Utilizing “Maslach Burnout Inventory” and “Teacher Sense of Efficacy Scale,” Savas et al. (2014) investigated the correlation between teacher self-efficacy and burnout among 163 teachers. Employing hierarchical multiple regression analysis, it was found that self-efficacy of teachers negatively correlated with their burnout experiences. In an EFL context, in Iran, Fathi et al. (2021) explored a structural model of teacher reflection, self-efficacy, burnout, and emotion regulation of EFL teachers. Employing confirmatory factor analysis, their findings indicted that teacher self-efficacy and teacher reflection negatively predicted the burnout of EFL teachers via the meditating role of emotion regulation. In another study, Bing et al. (2022) conducted a research to examine the predictive role of emotion regulation and self-efficacy in affecting EFL teachers’ burnout. Their results indicated both variables predicted teacher burnout significantly. Khani and Mirzaee (2015) explored the correlation among stressors, contextual variables, self-efficacy and burnout o EFL teachers in an Iranian context. Collecting data from a sample of 216 teacher, it was indicted that teacher self-efficacy was negatively related with burnout and reduced its negative effects. In another study, using structural equation modeling.

### 2.3. DTLP

Given the fact that love is intertwined and attached to humans’ nature and need for belongingness, giving a common definition for this concept has not an been easy task. However, some have tried to describe it. For instance, according to Berscheid (2006), “the word love is used in an astounding array of situations to describe an enormous range of attitudes, emotions, feelings, and behavior toward objects and people” (p. 172). As Loreman (2011) maintained, love is not merely limited to one’s private life and can be extended to his professional life, nature, and several more domains as well. As pointed out by Määttä and Uusiautti (2011), there are various categories of love, such as maternal and paternal love, romantic love, love for one another, the feeling of love toward an individual’s country, and pedagogical love. Despite its complicity and paradoxicality, pedagogical love is regarded as a vital construct of teaching (Maatta and Uusiautti, 2012). Teachers employing pedagogical love in their classrooms indicate that they love their students and have confidence in learners’ talent. Moreover, Pedagogical love does not merely focus on learners’ characteristics, rather it constantly tries to help them learn and develop as unique individuals (Hatt, 2005; Maatta and Uusiautti, 2012; Wilkinson and Kaukko, 2020). Pedagogical love pertains to teachers’ love toward their learners without expecting any rewards or favor in return (Skinnari, 2004; Johnson et al., 2019). It is worth noticing that pedagogical love not only facilitates students’ learning process but also help teachers to bond with their students in the classroom, motivating students to address their drawbacks while learning (Luguetti et al., 2019; Yin et al., 2019). The role of love in education can be traced back to early 16th century as various scholars and philosophers (e.g., Roger Ascham, John Locke and Martti Haavio) focused on this concept and discussed it. Martti Haavio’s perception was that it is better for teachers’ loving attitude can help develop learners’ personalities and promote their academic achievement (Määttä and Uusiautti, 2011). According to Loreman (2011), DTLP refers to the *passion, kindness, empathy, intimacy, bonding, sacrifice, forgiveness, acceptance, and community* between teacher and pupils and its theoretical framework is based on three categories, namely religious, psychological, and philosophical domains. As a well-known thinker on love in education, Paulo Freire indicates that it is not possible for teachers to teach without the courage to love, and that the existence of strong love for the world and for people is a necessity for education (McLaren, 1999; Madero, 2015; Freire et al., 2018). In line with the mainstream of positive psychology (Seligman, 2010), and the affective approaches in pedagogy, Barcelos and Coelho (2016) introduced the basic elements of DTLP, namely ethics, growth, care, respect, freedom, and dialog. It is worth pointing out that loving pedagogy can significantly contribute to learners’ emotional status, autonomy, motivation, academic achievement, and mental health (Darder, 2017; Ye et al., 2022). Extending this construct into the realm of SLA, Zhao and Li (2021) indicated loving pedagogy pertains to the kindness, affection, empathy, and care that teachers hold regarding the learners’ outcomes, feelings, needs, and learning process.

A review of the literature reveals that DTLP can play a vital role in affecting not only learners’ positive outcomes but also teachers’ well-being (e.g., Atmaca et al., 2020; Wilkinson and Kaukko, 2020; Kaukko et al., 2021; Derakhshan et al., 2022; Li and Miao, 2022; Wang et al., 2022). For instance, the results of the stud of Kaukko et al. (2021) indicated that teachers’ DTLP can be particularly conducive to refugee learners’ academic achievement and mental health. In this study, Barcelos (2020) demonstrated that DTLP in curriculum can lead to promoted motivation, achievement, well-being, autonomy, joy communication skills, engagement, resilience, and creativity of teachers and learners. In another study, Atmaca et al. (2020) investigated the association between teachers’ emotional, burnout and job satisfaction. To this end, 564 Turkish teachers participated in the study. Employing confirmatory factor analysis, their findings revealed that the love dimension positively predicted teachers’ job satisfaction. In addition, it was found that teachers’ feeling of love negatively predicted their burnout. By investigating the psychometric scales of Disposition toward Loving Pedagogy (DTLP), Teaching for Creativity Scale (TCS), and Utrecht Work Engagement Scale (UWES), Derakhshan et al. (2022) aimed at testing the relationship between teachers’ loving pedagogy, work engagement and teaching for creativity. Collecting data from a sample of 773 EFL teacher, their findings demonstrated that teaching for creativity and DTLP of teachers significantly predicted their work engagement.

### 2.4. The purpose of this study

**Figure 1:**
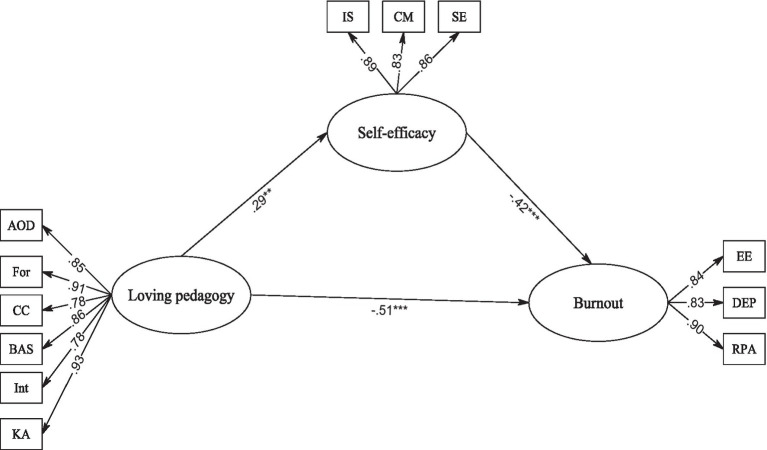
The final model of loving pedagogy, teacher self-efficacy, and burnout. ^*^p < 0.05; ^**^p < 0.01; ^***^p < 0.001.

The current study aimed to examine the relationship between DTLP, teacher self-efficacy, and teacher burnout among Chinese EFL teachers. Loving pedagogy, teacher self-efficacy, and teacher burnout are crucial factors for teacher well-being and effectiveness in the classroom. The above-mentioned studies can provide a groundwork for the investigation of the relationships between the variables, namely loving pedagogy disposition, self-efficacy and teacher burnout. Furthermore, given the fact that burnout can have a bearing on L2 learners’ academic achievement (Roohani and Dayeri, 2019), its role cannot be ignored in the field of EFL. however, what remains vague is the interplay of loving pedagogy, self-efficacy and teacher burnout. More specifically, to the best of our knowledge, there is no other study that has examined the association between these variables in a single study, particularly in L2 learning and teaching. Hence, as an attempt to fill this research lacuna, the current study advances the research on EFL teachers’ burnout by examining its potential relationships with loving pedagogy dispositions and teacher self-efficacy. According to the theoretical and empirical evidence mentioned above, a structural model regarding the correlations between the constructs (i.e., loving pedagogy dispositions, self-efficacy, and teacher burnout) was hypothesized (see Figure 1).

Based on extant empirical evidence (e.g., Skaalvik and Skaalvik, 2007, 2010; Fathi et al., 2021; Hassan and Ibourk, 2021; Bing et al., 2022) in which teacher self-efficacy negatively predicted burnout, it is hypothesized that teacher self-efficacy affects burnout negatively. Teacher self-efficacy, which refers to a teacher’s confidence in their ability to effectively perform their role and make a difference in the lives of their students, is a critical factor for teacher well-being and effectiveness. High levels of self-efficacy are associated with lower levels of stress and burnout, as well as improved job satisfaction and better performance. Also, loving pedagogy has been linked to positive outcomes in education, such as enhanced motivation, engagement, and academic achievement among students (Derakhshan et al., 2022; Wang et al., 2022). At the same time, loving pedagogy can also benefit teachers in terms of reducing stress and burnout. A teacher who adopts a loving pedagogy style is likely to feel more confident in their abilities and less stressed, as they are able to build positive and supportive relationships with their students (Atmaca et al., 2020; Zhao and Li, 2021). Accordingly, loving pedagogy is hypothesized to affect teacher burnout in a negative way.

Finally, following Seligman’s (2010) theoretical framework of positive psychology, it is hypothesized that loving pedagogy positively affects teacher self-efficacy. Although the literature linking DTLP and teacher self-efficacy is relatively scarce, there is a theoretical basis to suggest a potential link between the two constructs. The concept of loving pedagogy is concerned with a caring and supportive relationship between the teacher and the students (Noddings, 2012; Yin et al., 2019), which is expected to enhance teachers’ confidence in their ability to foster positive outcomes in their students. As a result, teachers who embrace loving pedagogy may feel more self-assured in their teaching abilities, leading to increased self-efficacy (Pekrun, 2021). In fact, previous studies have shown that teacher-student relationships characterized by care and trust are positively associated with teacher self-efficacy (Zhou et al., 2020; Cai and Tang, 2021). Therefore, it is plausible that DTLP could have a direct effect on teacher self-efficacy, although further empirical evidence is needed to establish this relationship more definitively.

Furthermore, it is hypothesized that teacher self-efficacy mediates the relationship between loving pedagogy and teacher burnout, such that higher levels of loving pedagogy lead to higher levels of self-efficacy, which in turn leads to lower levels of burnout. The mediating role of self-efficacy is justified on some grounds. First, empirical studies have found support for the mediating role of teacher self-efficacy in the relationship between instructional approaches and burnout. For example, a study by Yu et al. (2015) found that self-efficacy mediated the relationship between work stress and burnout. Similarly, a study by Cai et al. (2022) found that teacher self-efficacy mediated the relationship between professional community and teachers’ work engagement. Also, based on Bandura’s (1977) theory of self-efficacy, it is possible that loving pedagogy may be connected with mastery experiences, which could influence a teacher’s self-efficacy over time. A teacher who adopts a loving pedagogy style may feel more confident in their abilities and more likely to take on challenging tasks, leading to a sense of mastery and increased self-efficacy. Furthermore, research has shown that teacher self-efficacy is positively associated with teacher performance, job satisfaction, and well-being (Tschannen-Moran and Hoy, 2001; Klassen and Chiu, 2010). Teachers who believe in their ability to effectively manage their classrooms and promote student learning are more likely to experience lower levels of burnout and greater job satisfaction.

## 3. Materials and methods

The research design used in this research is quantitative research. Specifically, the researcher used a cross-sectional design to examine the relationships among the variables of interest, and collected data through an electronic survey comprised of valid questionnaires for the constructs of loving pedagogy, teacher self-efficacy, and teacher burnout. The study also used structural equation modeling (SEM) to test the hypothesized relationships among the variables.

### 3.1. Participants and setting

This online survey recruited 428 EFL teachers from various language academies and schools in China, including both private and public institutions. The institutions included primary, secondary and tertiary level schools. Convenience sampling was used to select participants, who were male (*n* = 196) and female (*n* = 232). The average age of participants was 26.32 years (SD = 6.94), and the average teaching experience was 6.83 years (SD = 3.04). The participants were distributed across different provinces in China, including both urban and rural areas. Specifically, 43% of the participants were from large cities, 28% were from medium-sized cities, and 29% were from smaller towns or rural areas. All participants had completed teacher training courses before participating in the study.

This research focused on EFL teachers in China, who face unique challenges in their teaching practice (Wu, 2001). EFL teachers work in a multicultural and diverse setting, where students may have varying levels of proficiency in the target language. They are required to create engaging and effective lesson plans while also ensuring that students meet language proficiency standards. Furthermore, EFL teachers often have large class sizes, limited resources, and little support from administration, which can contribute to their stress levels (Chen and Goh, 2011). Also, research evidence has suggested that EFL teachers are at a higher risk for burnout due to the demands of their job (Ghasemi, 2023), and the impact of burnout can be particularly concerning for EFL teachers, as it can affect their motivation, teaching effectiveness, and ultimately, student learning outcomes (Wang L., 2022; Wang Z., 2022).

### 3.2. Instruments

*The scales (TSES, MBI-ES, and DTLP) used in this study were all administered in English. Although the participants were EFL teachers in China, the items on the scales were understandable for them as they were all trained in English language teaching and had to demonstrate a certain level of proficiency in English in order to teach in their respective institutions/schools. Therefore, no translation was necessary for the administration of the scales in this study.*

#### 3.2.1. Teacher self-efficacy scale

Chinese EFL teachers’ self-efficacy perceptions were assessed using Teachers’ Sense of Efficacy Scale (TSES) designed by Tschannen-Moran and Hoy (2001). TSES constitutes 24 statements, rated on a Likert scale, which measure three underlying components including self-efficacy in instructional strategies (IS), classroom management (CM), and student engagement (SE). The respondents indicated their agreement level with each statement from1 (nothing) to 5 (a great deal). A sample item was “How much can you do to help your students think critically?” In this study, ω was 0.86 for this scale, showing its high reliability.

#### 3.2.2. Teacher burnout scale

Participants’ degree of burnout was gauged using Maslach burnout scale (MBI-ES), developed by Maslach et al. (1996). This self-report questionnaire consists of 22 statements assessing three facets of teacher burnout: emotional exhaustion (EE, 9 items), depersonalization (DEP, 5 items), and reduced personal accomplishment (RPA, 8 items). Every item is measured on 7-point Likert scale from 0 (never) to 6 (every day). MBI-ES proved to have acceptable reliability and validity indices (Maslach et al., 1996). A sample item of the scale is “I feel frustrated by my job.” The calculated ω for this scale was 0.82 in this study.

#### 3.2.3. Loving pedagogy scale

In order to measure EFL teachers’ loving pedagogy, Disposition toward Loving Pedagogy (DTLP) scale initially developed by Yin et al. (2019) was administered to the participants in this research. DTLP scale includes 29 statements which evaluate six dimensions of the construct: *acceptance of Diversity (AOD), Forgiveness (For)*, *Classroom Community (CC), Bonding and Sacrifice (BAS), Intimacy (Int), and Kind Acts (KA)*. The statements are rated on a 4-point Likert-scale from 1 (Strongly Disagree) to 4 (Strongly Agree). A sample item was “I make specific efforts to bond with students.” The reliability of this scale, as estimated with ω, was 0.85 in this research.

### 3.3. Procedure

The participants in this study were senior high school EFL teachers in China who were willing to participate in the study. The researcher initially reached out to a group of EFL teachers through professional networks and social media channels and invited them to complete the online survey. The teachers were also requested to forward the survey link to their colleagues via social apps such as WeChat and QQ, as well as through email, in order to cover a diverse range of participants. The online survey was hosted on a Chinese survey website^1^ and included three questionnaires on teacher self-efficacy, DTLP, and burnout. Prior to completing the survey, participants were provided with an explanation of the study’s purpose and instructions on how to fill out the survey and respond to the items. Participants were required to tick a box to indicate their voluntary participation and provide informed consent to take part in the survey. Additionally, the confidentiality of their collected information was emphasized to ensure the privacy and anonymity of their responses. The sample consisted of English teachers from various provinces and cities in China.

### 3.4. Data analysis

Descriptive statistics and correlations among the constructs were computed employing SPSS 21.0. The hypothesized model was tested with SEM using Mplus 7.0 program. I also used aggregated items for the observed indicators of each variable using a parceling approach procedure in SEM (Little et al., 2002). The advantage of using this approach instead of treating all items as indicators is the fact that parceling approach decreases the number of observed constructs in the model and enhances the parsimony of the model, leading to increased accuracy in testing the associations among the constructs (Little et al., 2002). As for estimating the latent constructs of teacher self-efficacy, burnout, and DTLP, the composite scores of their underlying components were parceled and considered as observed indicators (Joeng and Turner, 2015). More precisely, based on item-to-construct balance technique (Little et al., 2002), three parcels were created for each construct of teacher self-efficacy and burnout since each construct comprised three components. Accordingly, six parcels were also created for loving pedagogy construct. Concerning the reliability estimation of the used scales, McDonald’s Omega (ω) Coefficient (McDonald, 1999) was calculated. The single-common-method-factor approach (Podsakoff et al., 2003) was used to address the common method bias. As for evaluating the adequacy of the model fit, a number of goodness-of-fit indices were used: the comparative fit index (CFI), the Tucker-Lewis index (TLI), the root mean square error of approximation (RMSEA), and the standardized root mean square residual (SRMR). A model was considered to have good fit if CFI and TLI > 0.90, RMSEA < 0.06, and SRMR < 0.08 (Hu and Bentler, 1999). The power as well as significance of indirect effect of teacher self-efficacy was estimated using boot-strap procedure in which the sample under investigation was randomly resampled and replaced 1,000 times and then the indirect effect of 1,000 estimations was measured. In this approach, if the 95% confidence interval (CI) for an indirect effect fails to contain 0, the significance of the indirect effect is approved (MacKinnon et al., 2004).

## 4. Results

First, a single-common-method-factor technique was employed to test whether the three self-reported constructs (i.e., teacher self-efficacy, burnout, and DTLP) were influenced with common method bias (Podsakoff et al., 2003). To this end, the hypothesized three-factor model was compared against other alternative models (see Table 1). The comparisons demonstrated that the suggested three-factor model had a more satisfactory fit [χ^2^(25) = 46.379, CFI = 0.981, TLI = 0.980, RMSEA = 0.015, SRMR = 0.024]. As indicated in Table 1, the fit of the bifactor model (hypothesized three-factor + un-estimated common factor model) did not enhance substantially [| Δχ^2^ | (3) = 4.815, *p* = 0.57, ΔCFI = −0.001, ΔTLI = −0.001, ΔRMSEA = 0.008, ΔSRMR = −0.004]. Moreover, the hypothesized three-factor model demonstrated a substantially better fit than the other alternative models, highlighting that the common method bias influence was not significant in this research.

**Table 1:** Results of model fit for the common method bias test.

Models	_χ_2	*df*	Δχ^2^(Δ*df*)	CFI	TLI	RMSEA	SRMR
M1: single-factor model	274.124	28	227.745 (3)^***^	0.712	0.636	0.142	0.097
M2: two-factor model 1	193.719	27	147.340 (2)^***^	0.783	0.701	0.165	0.089
M3: two-factor model 2	137.271	27	90.892 (2)^***^	0.923	0.891	0.079	0.061
M4: two-factor model 3	162.576	27	110.704 (2)^***^	0.895	0.875	0.089	0.072
M5: hypothesized three-factor model	46.379	25	116.197	0.981	0.980	0.015	0.024
M6: bifactor model	41.564	22	−4.815(−3)	0.980	0.979	0.023	0.020

The hypothesized three-factor model was compared with all the alternative models. M2, two-factor model (loving pedagogy and burnout are combined); M3, two-factor model (teacher self-efficacy and burnout are combined); M4, two-factor model (loving pedagogy and teacher self-efficacy are combined); M6, Hypothesized three-factor + un-estimated common factor model ^***^p < 0.001.

Table 2 indicates the descriptive statistics as well as the correlations among the constructs. As expected, loving pedagogy was positively correlated with teacher self-efficacy and was negatively associated with teacher burnout. Additionally, teacher self-efficacy was negatively correlated with teacher burnout. Then, the structural model was tested with loving pedagogy as a predictor, teacher self-efficacy as a mediator, and teacher burnout as the criterion variable. It is noteworthy that gender, age, and teaching experience were controlled in this model testing. The results of model evaluation indicated that the model had a good fit to the data: χ^2^ (63) = 118.28, *p* < 0.001, CFI = 0.972, TLI = 0.65, RMSEA = 0.039, SRMR = 0.032. The path coefficients of the fit model are shown in Figure 1. As it was hypothesized, the direct effect of loving pedagogy on teacher burnout was significant (β = −0.51, *p* < 0.001). Loving pedagogy also had a positive effect on teacher self-efficacy (β = 0.29, *p <* 0.001), and teacher self-efficacy negatively affected teacher burnout (β = −0.42, *p* < 0.001). In addition, the indirect, mediating effect of teacher self-efficacy on burnout was also significant, although it should be noted that this was a partial effect (β = −0.12, *p* = 0.021, 95% CI [−0.06, −0.20]). While this effect was statistically significant at the *p* < 0.05 level, it was not a very strong path. Overall, the model explained 56.22% of the variance in teacher burnout based on the independent variables of loving pedagogy disposition and teacher self-efficacy.

**Table 2:** Descriptive statistics and correlations.

	*M* (SD)	1	2	3
(1) Teacher self-efficacy	3.76 (1.11)	1.00		
(2) Loving pedagogy	4.06 (0.91)	0.26^*^	1.00	
(3) Burnout	3.68 (0.92)	−0.36^**^	−0.48^**^	1.00

^*^p < 0.05; ^**^p < 0.01.

## 5. Discussion

The present research sought to examine the associations between DTLP, teacher self-efficacy, and teacher burnout among a sample of Chinese EFL teachers. The results of the SEM analysis indicated a significant negative effect of DTLP on teacher burnout, and teacher self-efficacy acted as a mediator in this relationship. These findings suggest that higher levels of DTLP are associated with greater levels of teacher self-efficacy, which in turn can reduce the risk of teacher burnout.

More specifically, firstly, it was revealed that the teachers’ self-efficacy beliefs significantly and negatively predicted burnout among EFL teachers. This finding accords with the findings of studies indicating that there is a strongly negative correlation between teacher self-efficacy and teacher burnout (e.g., Skaalvik and Skaalvik, 2007, 2010; Savas et al., 2014; Khani and Mirzaee, 2015; Ghasemzadeh et al., 2019; Fathi et al., 2021; Hassan and Ibourk, 2021; Bing et al., 2022). According to these studies, teachers believing in their own abilities in successfully completing particular teaching activities may have a strong effect on reducing their burnout. As Leiter and Schaufeli (1996) asserted, teachers with increased sense self-efficacy are more likely to be protected from feelings of apprehension and emotional exhaustion. Hence, it is postulated that teachers who are more confident in their capacities and competencies in conducting effective teaching strategies, managing their classrooms, and engaging the students, had less tendencies to feel reduced personal accomplishment, depersonalization, and emotional exhaustion. In addition, it can be argued that teachers with low level of perceptions of competence in classroom management are more likely to experience work-related stress, which in turn can give rise to both emotional exhaustion and depersonalization (Büssing and Glaser, 2000; Lloyd et al., 2017). Since work engagement is the exact opposite of burnout (Demerouti et al., 2010), this result is partly in accordance with that of Burić and Macuka (2018) who revealed that self-efficacy positively predicted work engagement off teachers.

Secondly, loving pedagogy was found to be a more powerful predictor of teacher burnout. This finding agrees with an earlier study that confirmed a significant positive impact of teacher love on teacher burnout (Atmaca et al., 2020). Put another way, it was found that teachers who subscribe to the pedagogy of love may feel less reduced personal accomplishment, depersonalization, and emotional exhaustion while teaching. These teachers often establish a sense of intimacy, create emotional bond with pupils, promote empathy in the classroom, and acknowledge the uniqueness of each learner (Derakhshan et al., 2022). One possible explanation for this finding is that loving pedagogy may enhance teacher self-efficacy and job satisfaction (Yin et al., 2019). When teachers establish emotional connections with their students, they may feel more effective in their teaching and experience a sense of fulfillment in their work (Grimmer, 2021). Moreover, by acknowledging the uniqueness of each learner, teachers may be more motivated to develop innovative and effective teaching strategies that meet the diverse needs of their students, resulting in their heightened job satisfaction (Loreman, 2011). Also, it is worth noting that passion, as a core component of a pedagogy of love can negatively predict all three components of burnout (i.e., emotional exhaustion, lack of personal accomplishment, and feelings of cynicism toward and detachment from work; Fernet et al., 2014). Furthermore, according to Vallerand et al. (2010), work passion is negatively correlated with one’s burnout. Therefore, one can argue that teachers who support DTLP are less prone to burnout while teaching. Another possible mechanism by which DTLP may reduce burnout is by fostering positive teacher-student relationships. Research has shown that positive relationships between teachers and students are associated with better academic and social–emotional outcomes for students (Roorda et al., 2011; Li et al., 2022). Moreover, positive teacher-student relationships can contribute to a positive school climate, which is associated with lower levels of teacher burnout (Roeser et al., 2013). Therefore, by promoting positive relationships with students, loving pedagogy may create a more supportive and positive work environment for teachers.

Finally, it was revealed that teacher self-efficacy mediated the relationship between loving pedagogy and teacher burnout. In other words, teachers who were equipped with DTLP were more likely to hold positive perceptions toward their abilities to teach in the class, which in turn helped them to have less probability of experiencing burnout. One explanation in this regard can be that the love that one has for something (i.e., his/her work) might be made use of to promote his interpersonal coping strategies and self-esteem (Newark et al., 2016). To further support this interpretation, it is worth noting that previous research has suggested that love is linked to greater levels of life satisfaction and psychological well-being (Kim and Hatfield, 2004; Lavy and Littman-Ovadia, 2011). Additionally, self-efficacy, or one’s belief in their ability to succeed in specific tasks or situations, has been shown to be positively correlated with well-being among teachers (Zee and Koomen, 2016; Ortan et al., 2021). Therefore, it is reasonable to hypothesize that teachers who practice loving pedagogy may also experience increased self-efficacy, which in turn could lead to greater work engagement. This idea is supported by the work of Newark et al. (2016), who found that one’s ability to love can have a positive impact on their self-efficacy.

## 6. Conclusions and implications

As demonstrated by the results, DTLP and self-efficacy of EFL teachers can lead to the alleviation of the probability of experiencing burnout among EFL teachers. The findings provide empirical evidence for the negative effect of loving pedagogy on teacher burnout and the mediating role of teacher self-efficacy. The outcomes emphasize the importance of promoting positive teacher dispositions, such as loving pedagogy, in order to support the well-being and effectiveness of foreign language teachers.

In the reviewed literature above, it was shown that DTLP can results in academic contexts, specifically in the context of EFL. As an attempt to propose a research agenda on the construct of loving pedagogy, the current study is innovative in that it sheds more light on this line of inquiry. Therefore, the findings of this study can have significant implications for L2 policy makers, teachers, and researchers. Additionally, given the fact that DTLP and self-efficacy are rooted in the domains of positive psychology (Costello and Stone, 2012; Derakhshan et al., 2022), EFL teacher education programs and practitioners should give proper attention to train pre-service teachers based on positive psychology framework which includes love, and self-efficacy as well as other psychological factors in order to promote the as wellbeing, leadership, and mental health of teachers. More specifically, EFL teacher education programs can emphasize the development of loving pedagogy dispositions and provide teachers with training in effective self-reflection and self-efficacy building strategies. For example, teacher education programs can encourage pre-service teachers to reflect on their personal beliefs and values, and how they can incorporate these into their teaching practice. They can also provide opportunities for pre-service teachers to observe and learn from experienced teachers who model loving pedagogy in their classrooms (Loreman, 2011). Furthermore, professional development programs can offer opportunities for practicing teachers to reflect on their own pedagogical practices and work on developing a positive and compassionate approach to teaching.

Workplace policies can also play a role in promoting a supportive work environment that values teacher well-being and encourages the development of positive teacher dispositions. Schools and educational institutions can create a culture that values and rewards positive teacher dispositions, such as loving pedagogy, and prioritize teacher well-being in their policies and practices (Yin et al., 2019). This can include providing resources for teachers to manage their workload and stress, as well as creating opportunities for teachers to collaborate and support each other in their professional development.

Although the findings of this study provided valuable insights into the interplay between DTLP, teacher self-efficacy, and teacher burnout, some limitations should be considered. First, the research was conducted with a sample of Chinese EFL teachers, and the results may not generalize to other populations of foreign language teachers. Moreover, multi-dimensional measures, such as the loving pedagogy scale, provide a more comprehensive assessment of the various dimensions of a construct as each dimension may have unique predictive value and could impact the overall relationship between constructs in the model. However, given the scope, purpose, and the sample size, the present researcher treated the measures as global constructs. Since the sample size was not sufficient to conduct analyses on multiple dimensions, analyzing a large number of dimensions with a small sample size could lead to issues with statistical power, which might have produced unreliable outcomes. As such, future researchers are invited to employ larger sample sizes and consider distinct dimensions of the used measures to identify which dimensions have the highest predictive value and can provide a more in-depth understanding of the associations between loving pedagogy, teacher self-efficacy, and burnout.

Furthermore, the researcher relied only on self-reported data, which may be subject to response bias and social desirability effects. Also, it is worth noting that while our findings suggest a positive impact of loving pedagogy on teacher burnout, it is important to acknowledge the potential bidirectional relationship between these constructs. It is possible that teachers who are experiencing burnout may be less likely to adopt a loving pedagogy approach, or that the stress of teaching may make it more challenging to maintain a loving disposition. Likewise, there may be a reciprocal interconnection between DTLP and teacher self-efficacy, where each concept positively influences the other. For instance, a teacher who has high levels of self-efficacy may be more likely to adopt a loving pedagogy style, as they feel confident in their ability to positively impact their students. Therefore, it is possible that the relationship between DTLP, teacher self-efficacy, and burnout is more complex than what is currently hypothesized, and future researcher might probe the potential bidirectional and reciprocal effects of these constructs.

In the same vein, in terms of the causality issue, it is important to note that the model tested in this study was a correlational model and could not imply causality. However, the theoretical framework underlying the study provided a logical basis for the hypothesized relationships between the constructs. It should be noted that the focus of this study was on the specific associations between DTLP, teacher self-efficacy, and teacher burnout. Future researchers could build on this study by considering additional constructs, such as social support, that could influence the relationships in the model.

Also, concerning the partial nature of the indirect effect through teacher self-efficacy as a mediator, it is important to note that although the indirect effect was significant at the *p* < 0.05 level, the effect size was relatively small. This suggests that while teacher self-efficacy does play a role in mediating the relationship between loving pedagogy disposition and teacher burnout, there may be other factors at play that also contribute to the development of burnout in teachers. In addition, it is possible that the effect size of the indirect effect was attenuated due to the fact that other variables were controlled in the structural model, such as gender, age, and teaching experience. As such, it might have been useful to conduct additional analyses to explore the role of these variables in the relationship between DTLP, teacher self-efficacy, and burnout. However, the present researcher acknowledges that the relatively weak correlation between DTLP and self-efficacy, particularly as compared to the stronger correlation between DTLP and burnout, deserves further exploration in future studies. Finally, the cross-sectional design of the study does not allow for causal inferences to be made, and further research is needed to establish the directionality of the relationships between the constructs.

## Data availability statement

The data analyzed in this study is subject to the following licenses/restrictions: the raw data supporting the conclusions of this article will be made available by the author, without undue reservation. Requests to access these datasets should be directed to SC, 154543459@qq.com.

## Ethics statement

The studies involving human participants were reviewed and approved by Basic Courses Teaching Department, Henan Judicial Police Vocational College, Zhengzhou. The patients/participants provided their written informed consent to participate in this study.

## Author contributions

The author confirms being the sole contributor of this work and has approved it for publication.

## Conflict of interest

The author declares that the research was conducted in the absence of any commercial or financial relationships that could be construed as a potential conflict of interest.

## Publisher’s note

All claims expressed in this article are solely those of the authors and do not necessarily represent those of their affiliated organizations, or those of the publisher, the editors and the reviewers. Any product that may be evaluated in this article, or claim that may be made by its manufacturer, is not guaranteed or endorsed by the publisher.

## Footnotes

^1^https://www.wjx.cn
